# Prevalence of and Factors Associated with Nephropathy in Diabetic Patients Attending an Outpatient Clinic in Harare, Zimbabwe: Methodological Issues

**DOI:** 10.4269/ajtmh.17-0011a

**Published:** 2017-09-07

**Authors:** Reza Pakzad, Saeid Safiri

**Affiliations:** Department of Epidemiology Faculty of Health Ilam University of Medical Sciences Ilam, Iran; Managerial Epidemiology Research Center Department of Public Health School of Nursing and Midwifery Maragheh University of Medical Sciences Maragheh, Iran and Department of Epidemiology and Biostatistics School of Public Health Tehran University of Medical Sciences Tehran, Iran E-mail: saeidsafiri@gmail.com

Dear Sir:

We have read the paper written by Machingura and others published in *The American Journal of Tropical Medicine and Hygiene* in December 2016.^1^ The authors aimed to evaluate the prevalence of and factors associated with nephropathy in a cross-sectional study of 344 diabetic patients in the capital of Zimbabwe. The prevalence of nephropathy was higher in two types of diabetes mellitus patients than previously reported. A considerable proportion of the participants had uncontrolled hypertension, obesity, and abnormal lipids, which are risk factors for cardiovascular disease. It was concluded that higher fructosamine and retinopathy were independent predictors of nephropathy. Although this study makes a notable contribution to the area, there are some methodological concerns that should be taken into account to avoid misinterpretations of study findings.

First, the authors used univariate analysis to examine the association of explanatory variables with nephropathy. The authors then created a single multivariate logistic regression analysis that included only variables with a very significant univariate *P* value (*P* < 0.01). More traditional model building comprises a systematic approach^2^ to more fully use the data and consider the impact of variables on each other, multicollinearity, effect modification, model fit, and model assumptions. For example, 1) the correlation between the explanatory variables is assessed to ensure that no multicollinearity exists between the studied explanatory variables. 2) Then, variables with a moderate *P* value, such as *P* < 0.2 in univariate analysis, are entered into the multivariable model and systematically assessed for model fit and model assumptions. In the study conducted by Machingura and others, only very significant explanatory variables (*P* < 0.01) were included into a single multivariate model. As a result, the multivariate model built by Machingura and others excluded explanatory variables with relatively smaller effect, even when statistically significant and/or biologically meaningful. In other words, the explanatory variables with large and small effect have been over- and underestimated in this multivariate model, respectively. This phenomenon has been referred to as “Testimation” bias.^3^ The findings of the aforementioned study may be biased due to the “Testimation” bias since key covariates were excluded from the model including *human immunodeficiency virus* status (*P* = 0.039), body mass index (*P* = 0.011), age (*P* = 0.144), triglycerides (*P* = 0.075), high-density lipoprotein cholesterol (*P* = 0.094), and hypertensive drugs (*P* = 0.120). The conclusion of independent effects of the two major associations cannot, therefore, be validated without additional analyses of these data. Furthermore, the accuracy and interpretability of one of these key findings, regarding fructosamine, is questionable, given the strange effect size reported of odds ratio = 1.00 (95% confidence interval = 1.00–1.01, *P* = 0.009).

Second, the authors stated that a questionnaire was applied to gather sociodemographic data, although many of these variables, including socioeconomic status, were not included in the univariate or multivariate analyses, despite their relevance to epidemiologic and clinical studies. Effective methods have been introduced to measure the socioeconomic status in epidemiological studies in low- and middle-income countries accurately.^4^

## References

[1] Machingura PI Chikwasha V Okwanga PN Gomo E, 2017 Prevalence of and factors associated with nephropathy in diabetic patients attending an outpatient clinic in Harare, Zimbabwe. Am J Trop Med Hyg 96: 477–482. 27994108 10.4269/ajtmh.15-0827 PMC5303056

[2] Hosmer DW Jr Lemeshow S Sturdivant RX, 2013 *Applied Logistic Regression.* Somerset, NJ: John Wiley & Sons.

[3] Steyerberg E, 2008 *Clinical Prediction Models: A Practical Approach to Development, Validation, and Updating*. New York, NY: Springer Science & Business Media.

[4] Howe LD Galobardes B Matijasevich A Gordon D Johnston D Onwujekwe O Patel R Webb EA Lawlor DA Hargreaves JR, 2012 Measuring socio-economic position for epidemiological studies in low-and middle-income countries: a methods of measurement in epidemiology paper. Int J Epidemiol 41: 871–886. 22438428 10.1093/ije/dys037 PMC3396323

